# Proprietary Preparations Falsely Described

**Published:** 1909-04-24

**Authors:** 


### PROPRIETARY PREPARATIONS FALSELY DESCRIBED.

The practice which has become so common among manufacturing druggists of attaching to some drug, more or less well known, a name which is registered as a trade-mark, has produced a great deal of confusion in the minds of the medical profession, amongst whose members many such products are "pushed" by enterprising travellers at exorbitant prices. It is well known now that aceto-salicylic acid, for example, is marketed by different firms under ten or a dozen different names, of which the two best known are perhaps aspirin and xaxa; and that the cost of this useful product, if ordered under its proper name, is about an eighth of that of its rival pseudonymous forms. Now this state of affairs is serious enough, but it is very largely the fault of medical men themselves that it has become so prevalent. If doctors will not take the trouble to sift the plausible tales of druggists' travellers, who are naturally intent on effecting a sale, a premium is put upon this form of covert mendacity. Hexamethylen-tetramin is another drug which has suffered severely in this way; its best-known alias is urotropin, and this again is simply the proprietary name under which the salt is sold by one particular firm.

All this, if not common knowledge, is at any rate understood by a large number of medical men. But a new and much worse form of this system has been brought to light, and one which involves positive misrepresentation of the most unprincipled kind. The method seems to be so far the monopoly of foreign—mainly German—firms who flood the profession in this country with pamphlets of apparently unimpeachable authority, and of most circumstantial evidence as to the value of their latest new preparations. Two papers on the matter in question from the *Therapeutische Monatschefte*, and the *Deutsche Medizinische Wochenschrift* are summarised in the *Medical Review*, from which we take many of the following particulars.

The feature common to most of the frauds, for they are nothing less, is the description of a mechanical mixture of two or more drugs as a new compound, whose alleged chemical formula and extremely lengthy constitutional name are disclosed. A short and catchy title, usually suggesting either the pretended action of the mixture, or some important constituent of it, is then given, under which protected name the article is sold to the profession and the public. Moreover many of the claims made on behalf of these drugs are false and unfounded to a degree, as is shown by examples which Professor Thoms and Dr. Harnack have published. Unfortunately there seems to be a considerable section of medical men in Germany who are willing to attach their names to testimonials, and enthusiastic clinical reports on the merits of these impostures, and in consequence the medical literature with which the advertisements bristle appears to confirm the statements of the manufacturers.

To take a few examples: arhovin, which has been most extensively advertised in Britain, has been described by the makers as diphenylamine thymyl benzoate. It has been shown, however, that no such acid as thymyl-benzoic exists; and that arhovin, which has been extolled as a urinary antiseptic, is a mechanical mixture of thymol, diphenylamine, and ethyl-ester of benzoic acid. By the same (German) firm two drugs known as jodofan and pyrenol have been extensively advertised. The former is said to act by giving off iodine-formol; the chemical formula allotted to it is an impossible one, it does not give off formaldehyde, and it contains about a tenth of the quantity of iodine disclosed by the formula assigned to it. The latter is an antipyretic, to which two most imposing names and formulæ have at different times been allotted; actually it is a mixture of salicylate and benzoate of soda with minute proportions of benzoic acid and thymol. Again, formurol has been introduced as a remedy for gout, and has been described as hexamethylen-tetramin sodio-citrate. As a matter of analysis it is a mechanical mixture of hexamethylen-tetramin (a product which burlesques under many protected names, of which urotropin is only one), with neutral and acid sodium nitrates. Phagocytin is sold in sealed vessels holding 1 c.c. each, and alleged to contain .05 gr. of pure nucleate of sodium, a contention completely upset by analysis.

There are many other foreign synthetic drugs, the claims made on behalf of which are equally fraudulent, but which we have not space here to deal with. The moral of these disclosures is surely obvious, though medical men in this country seem unaccountably slow in taking it to heart; even in some of the London teaching hospitals the visiting staff still order drugs of this sort under their proprietary names, and while that is so it is difficult to blame the students for acquiring the habit also. The commercial enterprise of German drug firms, together with the complacence of British medical men, has done a great deal of harm already, both to the public and to the profession. Self-drugging is directly encouraged whenever a doctor orders for a patient any patent proprietary drug, whether in compressed, fluid, pill, cachet, or any other form. Even when the prescriber is quite sure that he knows what it is he thus orders, the act is foolish; when his only source of information is the circular of a none too scrupulous firm of foreign druggists, it is positively immoral.

A healthy scepticism as to the merits of new synthetic drugs is not merely warranted by the experience of the past; it is the only proper scientific method by which that which is true may be distinguished from that which is false. The medical profession as a whole has been much too lax in accepting the fairy tales of the vendors of proprietary synthetic drugs, whether of British, Continental, or American origin. The goods thus sold are always very much more expensive under their trade aliases than they would be if ordered by their proper names; and, as has been shown, they are often barefaced frauds upon the consumer and the prescriber. Those who have got into the fatally easy habits of ordering synthetic products on the strength of the claims made for them by manufacturers would do well to read the two German papers from which we have quoted.

